# Early Vascular Developmental Toxicity and Underlying Mechanisms of 1-Bromo-3,6-dichlorocarbazole (1-B-36-CCZ) in Zebrafish Larvae

**DOI:** 10.3390/biology14060659

**Published:** 2025-06-06

**Authors:** Jie Gu, Ziyu Gong, Yue Fan, Jun Hu, Liguo Guo, Wenming Pei, Daqiang Yin

**Affiliations:** 1 Key Laboratory of Yangtze River Water Environment, Ministry of Education, College of Environmental Science and Engineering, Tongji University, Shanghai 200092, China; gujie@nies.org; 2 Nanjing Institute of Environmental Sciences, Ministry of Ecology and Environment, Nanjing 210042, China; gongziyu@nies.org (Z.G.); hujun@nies.org (J.H.); guoliguo@nies.org (L.G.); 3 Department of Toxicology, School of Public Health, Jiangsu Key Laboratory of Preventive and Translational Medicine for Geriatric Diseases, MOE Key Laboratory of Geriatric Diseases and Immunology, Suzhou Medical College of Soochow University, Suzhou 215123, China; 20235247076@stu.suda.edu.cn

**Keywords:** 1-Bromo-3,6-dichlorocarbazole, vascular toxicity, network toxicology, calcium signaling pathway

## Abstract

Polyhalogenated carbazoles (PHCZs) are emerging persistent organic pollutants that have attracted widespread attention due to their environmental occurrence and potential ecological risks. 1-Bromo-3,6-dichlorocarbazole (1-B-36-CCZ), which is a typical homolog of PHCZs produced as a byproduct in the dye industry, has been widely detected in various environmental media. In this study, we employed an integrated approach using an in vivo zebrafish model and network toxicology methods to systematically evaluate the vascular developmental toxicity of 1-B-36-CCZ and elucidate its underlying mechanisms. The experimental results revealed that the 96 h-LC_50_ of 1-B-36-CCZ in zebrafish larvae was 4.52 mg/L. Sublethal exposures (0.045–45 μg/L) significantly induced an increase in heart rate (*p* < 0.05) and an enlargement of the pericardial edema area (*p* < 0.01). Using *Tg(flk:eGFP)* transgenic zebrafish embryos to assess vascular toxicity at concentrations of 0, 0.045, 0.45, 4.5, and 45 μg/L, we observed that 1-B-36-CCZ exposure significantly reduced the length and anastomosis rate of intersegmental vessels (ISVs) at 30 hpf, and inhibited the development of the common cardinal vein (CCV) at 48 and 72 hpf as well as the subintestinal vessel (SIV) at 72 hpf. Quantitative PCR (qPCR) analysis further revealed that the expression of key angiogenic genes (*flk*, *kdr*, and *vegfa*) was significantly downregulated, thus corroborating the phenotypic observations. Moreover, a “compound–target–pathway” network model predicted that SRC kinase is a key molecular target for 1-B-36-CCZ action. Enrichment analysis of target protein-coding genes and verapamil replication experiments indicated that 1-B-36-CCZ may cause damage to early vascular development in zebrafish larvae by altering intracellular calcium ion content through the activation of the SRC-mediated calcium ion signaling pathway. This study provides new experimental evidence for elucidating the toxic mechanisms of PHCZ-type pollutants and offers a theoretical basis for environmental health risk assessments.

## Simple Summary

1-Bromo-3,6-dichlorocarbazole (1-B-36-CCZ) is a typical homolog of polyhalogenated carbazoles (PHCZs) that is widely present in various environmental media. In this study, we used a zebrafish model to evaluate the developmental and vascular toxicity of 1-B-36-CCZ. By integrating network toxicology methods, we found that the potential mechanism of 1-B-36-CCZ toxicity may involve the activation of the calcium signaling pathway via SRC kinase, thus leading to vascular toxicity. This potential mechanism was further validated by the efficacy of verapamil, a calcium channel blocker, in ameliorating the developmental abnormalities and vascular damage induced by 1-B-36-CCZ. These results provide new experimental data for elucidating the toxic mechanisms of PHCZ-type pollutants and offer a theoretical basis for environmental health risk assessments.

## 1. Introduction

Polyhalogenated carbazoles (PHCZs) represent a novel class of persistent organic pollutants [1] that is applied in various fields such as photoelectric materials, dyes, and pharmaceuticals [2]. Their primary sources include industrial waste and sewage discharge resulting from anthropogenic activities [3,4]. Owing to their persistent nature, these compounds can continuously exist and become widely distributed within aquatic environments, with detections reported in water bodies such as Lake Michigan in the United States and Taihu Lake in China [5,6,7]. Among these pollutants, 1-Bromo-3,6-dichlorocarbazole (1-B-36-CCZ) is a PHCZ that may be produced in industrial processes such as dye production [8]. Previous studies have reported that the maximum concentrations of 1-B-36-CCZ in the East China Sea reached 0.31 ng/L in seawater and 0.08 ng/g in sediments [8], while its concentration in drinking water from Wuhan was as high as 0.85 ng/L [9]. Given its persistence and bioaccumulative properties [10], 1-B-36-CCZ may pose potential environmental toxicities, although the current understanding of its ecotoxicological risks remains limited.

Structurally, PHCZs are similar to dioxin-like compounds and may exhibit dioxin-like toxicities, including developmental and cardiovascular toxicity [11,12,13]. For example, previous research has shown that 3-Bromocarbazole (3-BCZ) can induce developmental toxicity in zebrafish larvae [14]. 1,3,6,8-tetrabromo-9 H-carbazole (1368-BCZ) has been reported to affect angiogenesis in human umbilical vein endothelial cells by activating the hypoxia-inducible factor 1 (HIF-1) pathway and stimulating vascular endothelial growth factor (VEGF) production [15]. In another instance, 2,7-dibromocarbazole (2,7-DBCZ) induced pericardial edema malformation in Mugilogobius chulae, exerting significant effects on the cardiovascular system [16]. These studies collectively indicate that PHCZs can induce both developmental toxicity and cardiovascular toxicity. However, to the best of our knowledge, there have been no reported studies concerning the toxicity of 1-B-36-CCZ, thus making its toxicity evaluation both necessary and urgent.

Network toxicology has emerged as a promising discipline that provides a holistic view of the interactions between chemical compounds and biological systems through the lens of biological networks. This approach offers a novel paradigm for elucidating the molecular mechanisms underlying the adverse reactions induced by chemicals [17]. Molecular docking is often integrated with network toxicology to study the toxicity mechanisms of emerging contaminants [18]. Moreover, due to the transparency of zebrafish embryos, zebrafish are commonly used as model aquatic organisms for assessing pollutant toxicity [19]. Recent research has also utilized various transgenic models to determine organ-specific toxicity [20]. For instance, the transgenic zebrafish *Tg(flk:eGFP)* model allows for the direct visualization of in vivo angiogenesis, which aids in the investigation of vascular toxicity [21].

In this study, zebrafish were used as an experimental model organism to assess 1-B-36-CCZ-induced developmental and vascular toxicity, while the potential targets of 1-B-36-CCZ action were predicted and validated using a combination of cytotoxicity, molecular docking, polymerase chain reaction (PCR) assays, and recovery experiments. The findings of this study not only contribute to a better understanding of the mechanisms of toxicity of 1-B-36-CCZ but also provide the necessary toxicological data to support its regulatory management and risk assessment.

## 2. Materials and Methods

### 2.1. Chemicals and Reagents

1-Bromo-3,6-dichlorocarbazole (1-B-36-CCZ, CAS No: 100125-05-1) was purchased from MedChemExpress (MCE, Shanghai, China). Dimethyl sulfoxide (DMSO, CAS No. 67-68-5) was obtained from Solarbio Science & Technology Co., Ltd. (Beijing, China). SYBR^®^ Green Master Mix and the reverse transcription kit used were procured from Vazyme Biotech Co., Ltd. (Nanjing, China). Verapamil was supplied by Shanghai Maclin Biochemical Technology Co., Ltd. (Shanghai, China).

### 2.2. Zebrafish Culture and Husbandry

Wild-type AB zebrafish and the transgenic line *Tg(flk:eGFP)* were obtained from the Institute of Hydrobiology, Chinese Academy of Sciences (IHB, Wuhan, China). Adult zebrafish were housed in a recirculating water system maintained at 28 ± 0.5 °C under a 14 h light/10 h dark photoperiod and fed twice daily with brine shrimp. To collect fertilized eggs, males and females were placed at a 1:2 ratio in breeding tanks one day prior to spawning; the divider was removed the next morning under light to allow for natural spawning. Fertilized eggs were collected and examined under a microscope, and healthy embryos were selected for the subsequent exposure experiments. All animal procedures were performed in accordance with the guidelines for the care and use of laboratory animals and were approved by the Nanjing Institute of Environmental Science (Ethics No. IACUC-20240611) (Table S2).

### 2.3. Preparation of Solutions and Acute Exposure Assay

A total of 10 milligrams of 1-B-36-CCZ was dissolved in 1 mL of DMSO to prepare a stock solution of 10,000 mg/L. Subsequently, 100 μL of the stock solution was diluted with DMSO to a final volume of 1 mL, resulting in a working solution of 1000 mg/L. Serial dilutions were then performed to achieve the desired exposure concentrations. Based on pre-experimental data, an acute toxicity assay was conducted at concentrations of 0, 500, 1000, 2000, 4000, and 8000 μg/L. Each concentration was tested in triplicate with 10 eggs per replicate using 6-well plates over a 4-day continuous exposure period, during which embryo survival was monitored daily and dead embryos were removed and recorded.

### 2.4. Developmental Toxicity Testing of 1-B-36-CCZ

Based on the acute toxicity data (96 h-LC_50_ = 4.52 mg/L), subsequent toxicity studies were conducted at exposure ratios corresponding to 1/10,000, 1/1000, and 1/100. Normally developing zebrafish embryos at 4 h post-fertilization (hpf) were exposed to 1-B-36-CCZ at concentrations of 0.045, 0.45, 4.5, and 45 μg/L until 72 hpf, with three replicates per concentration and 20 embryos per replicate. Throughout the exposure, embryo survival and hatching were monitored daily. At 24 hpf, 15 embryos (5 per replicate) were randomly selected and imaged using a fluorescence stereomicroscope (Nikon SMZ25, Tokyo, Japan). At 72 hpf, 15 larvae (5 per replicate) were randomly chosen and similarly imaged (Nikon SMZ25, Tokyo, Japan). Image analysis and the quantification of the yolk sac area at 24 hpf, as well as larval body length, eye area, pericardial area, and heart rate at 72 hpf, were carried out using NIS-Elements D software (version 5.41.00; Nikon, Tokyo, Japan).

### 2.5. Acquisition of Fluorescent Images in Transgenic Zebrafish Larvae

For vascular imaging, *Tg(flk:eGFP)* transgenic zebrafish embryos were exposed to 1-B-36-CCZ at concentrations of 0.045, 0.45, 4.5, and 45 μg/L until 72 hpf. At specific time points (30 hpf, 48 hpf, and 72 hpf), 15 fluorescent embryos (5 per replicate) were randomly selected from each concentration group. Using a confocal high-content microscope, the vascular network was scanned and imaged in detail. At 30 hpf, the analysis focused on measuring the length of the intersegmental vessels (ISVs) as well as determining the congruence rate. At 48 hpf, images were assessed to quantify the area of the common cardinal vein (CCV) and to count the number of endothelial cells present. At 72 hpf, attention was directed toward evaluating the areas of both the subintestinal vessel (SIV) and the CCV. Image processing and quantitative analysis were performed using ImageJ1.x software.

### 2.6. Verapamil Recovery Experiment

Based on previous findings that 0.5 mg/L verapamil can significantly mitigate the increase in zebrafish myocardial contractility induced by elevated calcium ion concentrations [22], a verapamil concentration of 0.5 mg/L was employed in the recovery experiment. This study included four groups: a control group, a 1-B-36-CCZ exposure group, a co-exposure group comprising both 1-B-36-CCZ and verapamil (recovery group), and a verapamil-only group. After an exposure period of 72 hpf, 15 zebrafish larvae were randomly selected from each group and imaged using a fluorescence stereomicroscope (Nikon SMZ25, Tokyo, Japan). Pericardial area and heart rate measurements were subsequently obtained from these images using NIS-Elements D software (version 5.41.00; Nikon, Tokyo, Japan). Additionally, vascular images of the 72 hpf *Tg(flk:eGFP)* larvae were acquired using a confocal high-content microscope, and the SIV area was measured using ImageJ1.x.

### 2.7. Construction of 1-B-36-CCZ and Vascular Injury Targets

To predict the potential targets of 1-B-36-CCZ, the SMILES string of the compound was input into the SwissTarget Prediction database [23] with the species set to Homo sapiens. The predicted targets were then downloaded. In parallel, the disease-related targets associated with vascular injury were retrieved from the Genecards database [24] by searching for the keyword “vascular injury”. Overlapping targets between those predicted for 1-B-36-CCZ and those associated with vascular injury were subsequently identified using the Venn diagram tool [25], with the intersecting genes considered the potential targets for 1-B-36-CCZ-induced vascular injury.

### 2.8. PPI Network Construction and Enrichment Analysis

Protein–protein interaction (PPI) networks were constructed using the STRING database [26] and visualized in Cytoscape software (v3.10.3). The intersecting genes from the targets associated with 1-B-36-CCZ-induced vascular injury were imported into STRING with the species set to human. The resulting interactions were then exported into Cytoscape to generate a comprehensive PPI network. Core targets were identified as those exhibiting a degree value at least twice the median of the network. To elucidate significant pathways involved in the vascular injury induced by 1-B-36-CCZ, KEGG pathway enrichment analysis was performed on the target genes using the DAVID database [27,28].

### 2.9. Molecular Docking

Molecular docking was employed to further analyze the binding interactions and affinities between 1-B-36-CCZ and the selected core target proteins. Initially, appropriate ligand structures for Homo sapiens were obtained from the PDB database [29], ensuring that the resolution of the chosen structures was greater than 2.0. The ligand molecules were processed using AutoDockTools-1.5.6 to add hydrogens and remove water molecules, and the resulting structures were saved in the pdbqt format. In parallel, the target protein structures were cleaned using PyMOL 2.3.4, during which water molecules and co-crystallized ligands were removed. These processed receptor structures were then converted to pdbqt format using AutoDockTools-1.5.6. Docking calculations were executed through CMD command lines within AutoDockTools-1.5.6, and the results detailing the binding conformations and corresponding affinities were visualized and analyzed using Discovery Studio 2024 and PyMOL 2.3.4.

### 2.10. QPCR

At 144 hpf following exposure to 1-B-36-CCZ, 50 zebrafish larvae per replicate (a total of 150 larvae per concentration) were randomly selected for total RNA extraction using Trizol reagent. cDNA was synthesized using the PrimeScript^®^ RT kit. A quantitative fluorescence PCR was then performed using SYBR^®^ Green on a CFX Connect Real-Time System (Bio-Rad, California, USA) to determine the relative expression levels of vascular development-related genes (*flk*, *kdr*, and *vegfa*) as well as genes involved in the calcium ion signaling pathway (*erbb2*, *orai1*, *slc8a1*, and *htr6*). β-actin was used as an internal control, and the 2^–ΔΔCt^ method was applied to calculate relative expression levels. Primer sequences are listed in Supplementary Table S1.

### 2.11. Data Analysis

Statistical analyses were conducted using GraphPad Prism 8 (GraphPad Software, San Diego, CA, USA). Data normality was evaluated using the Kolmogorov–Smirnov test. A one-way analysis of variance (ANOVA) was performed, followed by Tukey’s post hoc test to assess differences among groups. A *p*-value less than 0.05 was considered statistically significant. Significant differences between the exposure and control groups were indicated as follows: *p* < 0.001 (***), *p* < 0.01 (**), and *p* < 0.05 (*). Meanwhile, differences between the exposure and recovery groups were expressed as follows: *p* < 0.001 (^###^), *p* < 0.01 (^##^), and *p <* 0.05 (^#^).

## 3. Results

### 3.1. Effects of 1-B-36-CCZ Exposure on Acute Toxicity and General Development in Zebrafish

**Figure 1:**
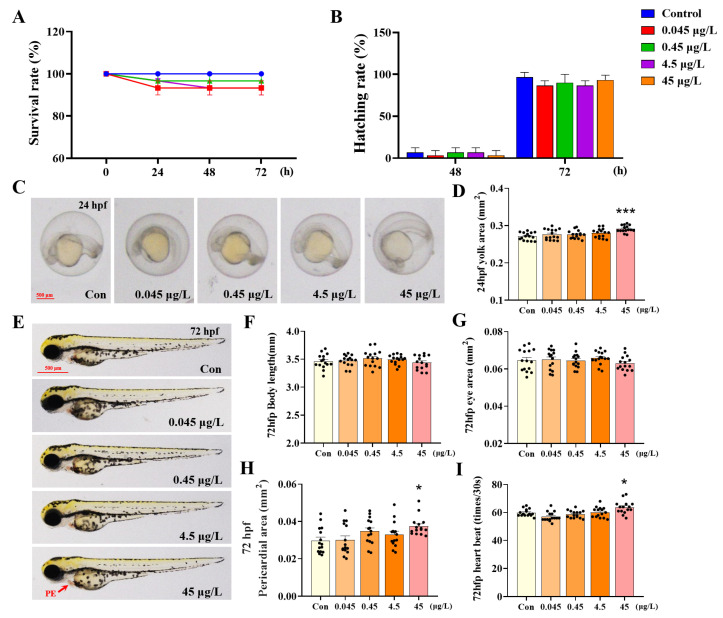
Effects of 1-B-36-CCZ exposure on acute toxicity and general development in zebrafish. Statistical graph of (**A**) survival rate and (**B**) hatching rate of zebrafish embryos after 72 h of 1-B-36-CCZ exposure. (**C**) Images of zebrafish embryos after 24 h of 1-B-36-CCZ exposure. Statistical graph of (**D**) yolk sac area in embryos. (**E**) Images of zebrafish larvae after 72 h of 1-B-36-CCZ exposure. Statistical graph of (**F**) body length, (**G**) eye area, (**H**) pericardial area, and (**I**) heart beat of 72 hpf zebrafish larvae. * *p* < 0.05, *** *p* < 0.001.

Acute toxicity tests indicated that the 96 h-LC_50_ of 1-B-36-CCZ in zebrafish larvae was 4.52 mg/L (Figure S1). In general developmental toxicity tests, no significant differences in survival or mortality rates were observed between the control group and the groups exposed to various concentrations of 1-B-36-CCZ (Figure 1A,B). At 24 hpf, compared with the control group, exposure to 45 μg/L of 1-B-36-CCZ resulted in a significant increase of 7.37% in the yolk sac area (Figure 1C,D, *p* < 0.001). By 72 hpf, no significant changes were detected in the body length or eye area of zebrafish larvae compared with the control group (Figure 1E–G). In contrast, both pericardial area and heart rate were significantly increased by 25.66% and 6.35%, respectively (Figure 1H,I, *p* < 0.05). These results indicate that 1-B-36-CCZ adversely affects early developmental processes in zebrafish larvae.

### 3.2. Effects of 1-B-36-CCZ on Vascular Development in Zebrafish Larvae

**Figure 2:**
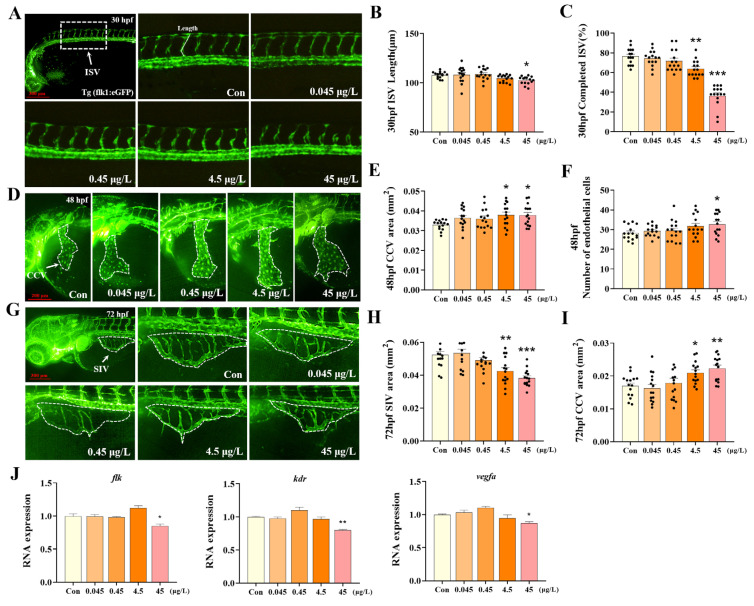
Effects of 1-B-36-CCZ on vascular development in zebrafish larvae. (**A**) Fluorescent images of ISV in zebrafish larvae after 30 h of 1-B-36-CCZ exposure. Statistical analysis of (**B**) length of ISV and (**C**) completed rate of ISV. (**D**) Fluorescent images of CCV in zebrafish larvae after 48 h of 1-B-36-CCZ exposure. (**E**) Statistical analysis of the area of CCV and (**F**) number of endothelial cells. (**G**) Fluorescent images of SIV in zebrafish larvae after 72 h of 1-B-36-CCZ exposure. (**H**) Statistical analysis of the area of SIV and (**I**) CCV. (**J**) Expression levels of vascular development-related genes, *flk, kdr*, and *vegfa*. * *p* < 0.05, ** *p* < 0.01, *** *p* < 0.001.

Transgenic *Tg(flk:eGFP)* embryos were exposed to 1-B-36-CCZ at concentrations of 0, 0.045, 0.45, 4.5, and 45 μg/L until 72 hpf. At 30 hpf (Figure 2A), the ISV length in the 45 μg/L exposure group was significantly reduced by 5.01% compared with the control group (Figure 2B, *p* < 0.05). Moreover, the ISV matching rate was significantly decreased by 16.48% and 52.14% in the 4.5 and 45 μg/L exposure groups, respectively (Figure 2C, *p* < 0.01). At 48 hpf (Figure 2D), the area of the CCV in the 4.5 and 45 μg/L exposure groups was significantly increased relative to the control (Figure 2E, *p* < 0.05), and the number of endothelial cells in the CCV was significantly higher in the 45 μg/L group (Figure 2F, *p* < 0.05). At 72 hpf (Figure 2G), compared with the control, the SIV area was significantly reduced by 18.94% and 27.02% in the 4.5 and 45 μg/L exposure groups, respectively (Figure 2H, *p* < 0.01), whereas the CCV area was significantly increased by 21.90% and 31.68% (Figure 2I, *p* < 0.05). In addition, the expression levels of vascular development-related genes (*flk*, *kdr*, and *vegfa*) were significantly suppressed (Figure 2J, *p* < 0.05), thus further demonstrating that 1-B-36-CCZ exposure impairs vascular development in zebrafish.

### 3.3. Potential Targets and Signaling Pathways Involved in 1-B-36-CCZ-Induced Vascular Toxicity

**Figure 3:**
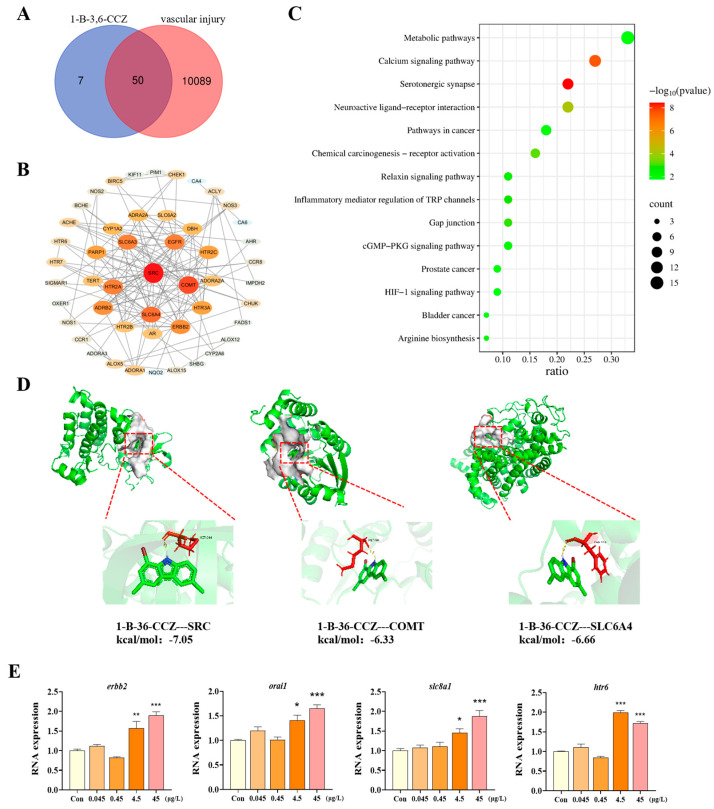
Potential targets and signaling pathways involved in 1-B-36-CCZ-induced vascular toxicity. (**A**) Venn diagram of potential common targets. (**B**) Diagram of PPI network. (**C**) KEGG enrichment pathway map of potential targets. (**D**) Molecular docking of 1-B-36-CCZ and SRC, COMT, and SLC6A4. (**E**) Expression levels of calcium signaling pathway-related genes (*htr6*, *orai1*, *erbb2*, and *slc8a1*). * *p* < 0.05, ** *p* < 0.01, *** *p* < 0.001.

Using the SwissTarget Prediction database, 57 targets related to 1-B-36-CCZ were identified, while 10,139 targets associated with vascular toxicity were retrieved from the Genecards database. The Venn diagram tool was then used to screen and identify 50 potential common targets for further analysis (Figure 3A). An analysis of these 50 targets using the STRING database, followed by visualization with Cytoscape software, showed that there was a protein–protein interaction (PPI) network consisting of 50 nodes and 142 edges, with an average node degree of 5.68. Network analysis revealed that the top three targets, based on relevance, were SRC, COMT, and SLC6A4, with SRC identified as the core target (Figure 3B). The molecular docking of 1-B-36-CCZ with SRC, COMT, and SLC6A4 revealed binding energies of −7.05 kcal/mol, −6.33 kcal/mol, and −6.66 kcal/mol, respectively. The lowest binding energy observed with SRC indicates the strongest interaction with 1-B-36-CCZ.

To further explore the potential toxicological pathways and underlying mechanisms, KEGG pathway enrichment analysis was performed on these 50 targets using the DAVID database. A statistically significant bubble chart was generated that visually ranked the top 14 KEGG pathways based on their *p*-values. Among these pathways, the serotonergic synapse and calcium signaling pathways were the most significant (Figure 3C). PCR analysis further demonstrated that exposure to 1-B-36-CCZ significantly upregulated the expression of calcium signaling pathway-related genes (*erbb2*, *orai1*, *slc8a1*, and *htr6*), thus confirming that 1-B-36-CCZ perturbs the calcium signaling pathway.

### 3.4. Verapamil Alleviates 1-B-36-CCZ-Induced Vascular Toxicity

**Figure 4:**
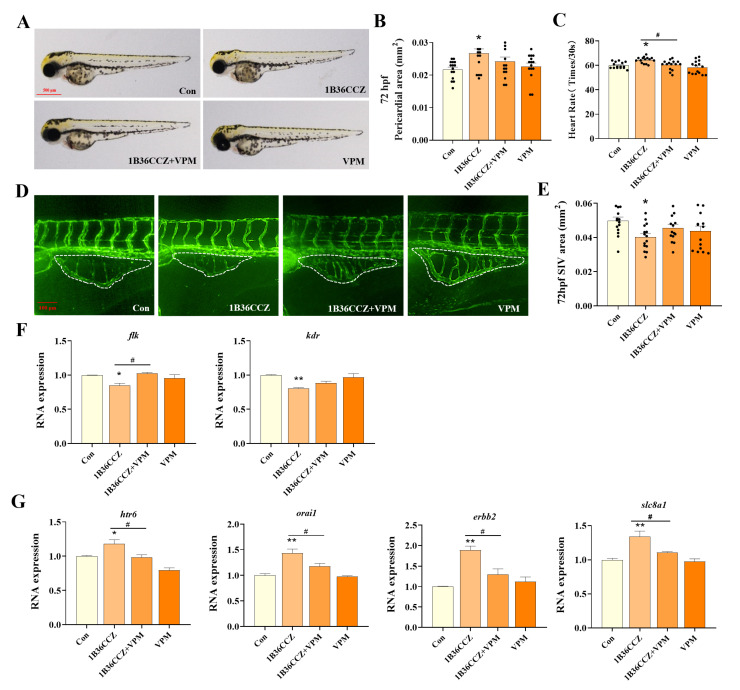
Verapamil alleviates 1-B-36-CCZ-induced vascular toxicity. (**A**) Images of zebrafish larvae after 72 h of recovery from exposure. Statistical graph of (**B**) pericardial area and (**C**) heart beat of 72 hpf zebrafish larvae. (**D**) Fluorescent images of SIV (the part circled by a white dashed line) in zebrafish larvae after 72 h of recovery exposure. (**E**) Statistical analysis of the area of SIV. (**F**) Expression levels of vascular development-related genes, *flk*, and *kdr.* (**G**) Expression levels of calcium signaling pathway-related genes (*htr6*, *orai1*, *erbb2*, and *slc8a1*). *^/#^ *p* < 0.05, ** *p* < 0.01.

To investigate whether the calcium channel blocker verapamil could mitigate the developmental and vascular toxicity induced by 1-B-36-CCZ, zebrafish were co-exposed to 0.5 mg/L verapamil and 45 μg/L 1-B-36-CCZ. At 72 hpf, compared with the 1-B-36-CCZ exposure group, zebrafish co-exposed to 1-B-36-CCZ and Verapamil showed a trend toward a reduced pericardial area and a significant decrease in heart rate, such that these parameters were not significantly different from those of the control group (Figure 4A–C). Similarly, the SIV area of *Tg(flk:eGFP)* zebrafish larvae in the co-exposure group exhibited a partial recovery (Figure 4D,E, *p* < 0.05). The PCR results further revealed that, compared with the 1-B-36-CCZ group, the expression of vascular development-related genes (*flk* and *kdr*) in the recovery group tended to be upregulated (Figure 4F, *p* < 0.05), whereas the expression levels of calcium signaling pathway-related genes (*htr6*, *orai1*, *erbb2*, and *slc8a1*) were significantly decreased (Figure 4G, *p* < 0.05).

## 4. Discussion

1-B-36-CCZ is a type of PHCZ with strong persistence that is widely distributed in the environment [11]. Because its structure is similar to that of dioxin-like compounds, 1-B-36-CCZ may exhibit dioxin-like toxicity [30]. To date, few studies have addressed its toxicological effects. Zebrafish are frequently used to evaluate pollutant toxicity, as they are important experimental animal models [31,32]. In this study, we employed a zebrafish model combined with network toxicology to conduct a preliminary investigation of the toxic effects induced by 1-B-36-CCZ and to explore its potential mechanisms.

The yolk sac is the initial site of hematopoiesis in many animals; interference with its function may directly affect embryonic growth and development, thus leading to malformations, tissue damage, or even embryonic death [33]. Our results showed that, a significant increase in yolk sac area was observed at 24 hpf, even at concentrations of 1-B-36-CCZ that do not affect zebrafish larval survival or hatching. By 72 hpf, both the pericardial area and the heart rate of zebrafish larvae were significantly increased. Previous studies have demonstrated that 2,7-DBCZ exhibits developmental toxicity and cardiac teratogenicity [34], which is consistent with our findings and indicates that 1-B-36-CCZ exposure adversely affects the early development of zebrafish larvae. Since heart rate and pericardial area are sensitive sublethal endpoints used to assess cardiovascular development [35], they serve as valuable indicators of cardiovascular status. Moreover, earlier research has also suggested that 1368-BCZ possesses potential vascular toxicity in zebrafish [15]. These observations warrant further investigation into the effects of 1-B-36-CCZ on the vascular development of zebrafish larvae.

ISVs are vital vascular structures formed during zebrafish embryogenesis, connecting the dorsal aorta (DA) and the posterior cardinal vein (PCV) [36], and they typically anastomose around 30 hpf [37], thus playing a central role in capillary blood circulation between the aorta and somites [38]. The CCV plays a key role in the cardiovascular system by transporting blood from the posterior region back to the heart [38], while the SIV constitutes a major arterial component of the zebrafish intestinal vasculature [39]. The transgenic zebrafish line *Tg(flk:eGFP)* enables the specific visualization of the vascular network due to the endothelial expression of EGFP driven by the flk1 promoter, and it has been widely used in vascular development research [40]. In our study, *Tg(flk:eGFP)* zebrafish were used to assess the effects of 1-B-36-CCZ on early vascular development. Our findings showed that exposure to 1-B-36-CCZ significantly inhibited the development of ISVs, the CCV, and the SIV. In addition, the expression of key genes associated with vasculogenesis and endothelial cell proliferation, including *flk*, *vegfa*, and *kdr*, was significantly downregulated, thus further confirming that 1-B-36-CCZ induces vascular toxicity in zebrafish larvae.

Environmental pollutants often cause complex network toxicology by disrupting various biomolecules. Traditional toxicological methods are often insufficient to comprehensively and systematically assess the interrelationships among different toxic effects [17]. In contrast, network toxicology integrates vast amounts of bioinformatics data and big data analytical techniques, thus allowing for a more comprehensive and systematic analysis of the intricate networks between toxic compounds and biological systems [41]. Molecular docking can be used to study how drugs modulate signaling pathways by binding to key targets [42]. In this study, targets related to both 1-B-36-CCZ and vascular toxicity were separately screened using the SwissTarget Prediction and Genecards databases, and common targets were identified via Venn diagram analysis. By constructing a PPI network using the STRING platform and Cytoscape software, combined with the results of molecular docking experiments, SRC was found to be the potential key target most closely associated with 1-B-36-CCZ-induced vascular toxicity. As a member of the Src family kinases (SFKs), SRC participates in various cellular events through the phosphorylation of L-type calcium channels (Cav1.2) and can modulate VEGF receptor signaling [43], which is critical for vascular development [44]. Furthermore, KEGG enrichment analysis revealed the significant enrichment of the calcium signaling pathway, which plays a central role in both the physiological and pathological processes of the cardiovascular system [45]. The gene *htr6*, belonging to the G protein-coupled receptor (GPCR) family and functioning as a receptor for serotonin, can induce increases in intracellular calcium levels [46]. In addition, *erbb2*, a receptor tyrosine kinase of the ErbB family, is highly expressed in the vasculature and heart, influences VEGF receptor signaling, and is essential for angiogenesis [47,48]. The ErbB receptor signaling pathway is also regulated by calmodulin (CaM) [49]. Previous studies have indicated that SRC and the calcium signaling pathway form a bidirectional regulatory network that jointly participates in processes such as cell proliferation, migration, and immune response [50]. Furthermore, SRC/ERBB2 can mediate the MAPK/ERK signaling pathway to inhibit proliferation and induce apoptosis [51]. The protein encoded by *orai1* is a calcium ion channel on the cell membrane and is involved in the inward flow of calcium ions. When the levels of the *orai1* gene expression are increased, the influx of calcium ions from outside the cell into the cell is promoted, thereby increasing intracellular calcium ion content [52]. The increased expression levels of *slc8a1a* enhance calcium ion inward flow and regulate intracellular calcium ion concentration [53]. Our results showed that following 1-B-36-CCZ exposure, the expression levels of calcium signaling pathway-related genes (*erbb2, orai1, slc8a1a*, and *htr6*) were significantly upregulated, thus suggesting that 1-B-36-CCZ may inhibit vascular development by increasing intracellular calcium levels and reducing VEGF receptor signaling.

Verapamil, a calcium channel blocker, reduces calcium influx into cells and is commonly used in the treatment of cardiovascular diseases [22,54]. In our study, the co-exposure of zebrafish larvae to verapamil and 1-B-36-CCZ was employed to explore whether inhibiting calcium entry could alleviate the developmental and vascular toxicities induced by 1-B-36-CCZ. The results demonstrated that, compared with the 1-B-36-CCZ exposure group, the verapamil recovery group exhibited reductions in pericardial area and heart rate, as well as a partial restoration in SIV area and the expression levels of vascular development-related genes (*flk* and *kdr*). Additionally, alterations in the expression of calcium signaling pathway-related genes (*htr6*, *orai1*, *erbb2*, and *slc8a1a*) indicate that verapamil effectively impacts the calcium signaling pathway. These findings further confirm that 1-B-36-CCZ induces early vascular developmental impairment in zebrafish larvae through the modulation of intracellular calcium and the suppression of VEGF receptor signaling. However, it is important to point out that the present study has some limitations. In particular, we were not able to perform systematic in vitro experimental validation, which limits our in-depth understanding and corroboration of these mechanisms. Second, the experimental data were mainly obtained from short-term exposure experiments, which may not fully reflect the risks associated with long-term low-dose exposures under real environmental conditions. Therefore, future studies should include long-term exposures at multiple concentration gradients and integrate multi-omics approaches, such as metabolomics and transcriptomics, to establish a more comprehensive dose–response prediction model.

## 5. Conclusions

In conclusion, our study systematically elucidated the toxic effects of 1-B-36-CCZ on the cardiovascular system and its potential molecular mechanisms by using a zebrafish model combined with network toxicology, molecular docking, and PCR techniques. Our results showed that 1-B-36-CCZ could trigger vascular developmental abnormalities through the calcium signaling pathway. The present study further verified the above conclusion via a verapamil recovery assay. This study can contribute to a more comprehensive assessment of the environmental health risks posed by this compound and provide valuable insights for the systematic evaluation of the toxicology of emerging environmental pollutants.

## Data Availability

The raw data supporting the conclusions of this article will be made available by the authors upon request.

## Supplementary Materials

The following supporting information can be downloaded at https://www.mdpi.com/article/10.3390/biology14060659/s1, Table S1: Primer sequences for quantitative real-time polymerase chain reaction; Figure S1: Statistical plot of mortality data of zebrafish larvae after 96 h of exposure to 1-B-36-CCZ. * *p* < 0.05, ** *p* < 0.01, *** *p* < 0.001; Table S2: Affdavit of Approval of Animal Ethical and Welfare.
