# Supplementary material for: Early Vascular Developmental Toxicity and Underlying Mechanisms of 1-Bromo-3,6-dichlorocarbazole (1-B-36-CCZ) in Zebrafish Larvae
Source: Biology (Basel). 2025 Jun 6;14(6):659. doi: 10.3390/biology14060659 (PMC12189126; doi:10.3390/biology14060659)
Supplement: Supplementary file 1 [file biology-14-00659-s001.zip › biology-3641859-supplementary.pdf]

Supplementary Materials

Table S1. Primer sequences for quantitative real-time polymerase chain reaction.

Accession number		Forward	Reverse
NC_007134.7	*htr6*	5’-CCTTGGCTTCTGGCTGTCAT-3’	5’-ACCAGTGCCACCATCAAGTC-3’
NC_133185.1	*orai1*	5’- GTGAGTCTCCGCGCTGTTTA-3’	5’- CCGGCGTTTTGTTTCTCGTG-3’
NC_133186.1	*slc8a1a*	5’- AGGAGGAGGAGGAGGAGGAG -3’	5’- TCCCTCCCTCCCTCCCTCCCT -3’
NC_007123.7	*erbb2*	5’- GTACTGGGCCCAAAGACTCC-3’	5’- GTCGTAGATAGTGGGCGGTG-3’
NC_007127.7	*vegfa*	5’-ATGAGAACCACACAGGACGG-3’	5’-ACACTCTCGCTTTGCTTCCT-3’
NC_007125.7	*flk*	5’- TCACAACTGGATGGATTTGG -3’	5’- GCCGACAGTCTTTTCTTTGC -3’
NC_007131.7	*kdr*	5’- CTTGGCAGCCAGAAATATCC -3’	5’-GACGAGCATCTCCTTTACGG-3’
NM_181601.5	*β-actin*	5'- ACAGGGAAAAGATGACACAGATCA -3'	5'- CAGCCTGGATGGCAACGTA -3'

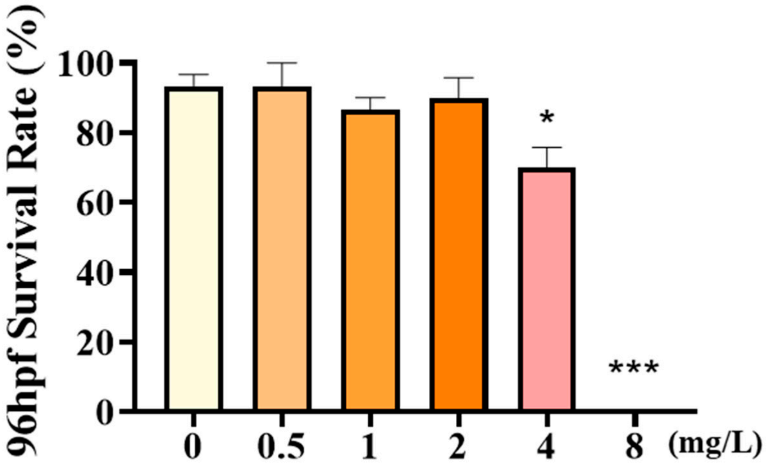

Figure S1. Statistical plot of mortality data of zebrafish larvae after 96h of exposure to 1-B-36-CCZ. ***P < 0.05, **P < 0.01, ***P < 0.001*.**

Table S2: Affdavit of Approval of Animal Ethical and Welfare

Affidavit of Approval of Animal Ethical and Welfare

Approval No.	IACUC-20240611

The animal use protocol listed below has been reviewed and approved by the Animal Ethical and Welfare Committee (AEWC), hereby certify.

Protocol Title	Research on the Toxic Effects and Mechanisms of Polyhalogenated Carbazoles (PHCZs) on Zebrafish				
Applicant	Jie Gu	Title/Degree	Assistant researcher	Email	gujie@nies.org
Principle Investigator (PI)	Guixiang Ji	Title/Degree	Researcher	Email	jgx@nies.org
Institution	Nanjing Institute of Environmental Sciences, Ministry of Ecology and Environment				
Species or Strains	Zebrafish (*Danio rerio*)			Quantity	3000 embryos
Period of Protocol	2024/6/11 — 2025/2/11		Application date	2024/6/11	
Number of Animal use permit	SYXK（Su）：2024-06-11				
Results of inspection	☑ Agree.				
Chief Facility Officer	Gie Feng			Date	2024-06-11
Supplement		Stamp: 生态环境部 农药环境评价与污染控制重点实验室			
